# Penicillin allergy documentation in the UAE: 10-year trends and stewardship implications

**DOI:** 10.3389/fmed.2026.1891395

**Published:** 2026-08-06

**Authors:** Aaesha Alteneiji, Saif Al-Shamsi, Farida Almarzooqi

**Affiliations:** 1 Department of Internal Medicine, College of Medicine and Health Sciences, United Arab Emirates University, Al Ain, United Arab Emirates; 2 Allergy and Clinical Immunology, Department of Internal Medicine, Tawam Hospital and Sheikh Tahnoun Bin Mohammed Medical City (STMC), Al Ain, United Arab Emirates

**Keywords:** allergy de-labelling, antimicrobial stewardship, beta-lactam allergy, penicillin allergy, United Arab Emirates

## Abstract

**Introduction:**

Penicillin allergy labels are frequently documented in electronic medical records (EMRs), although most patients are not truly allergic upon formal evaluation. Inaccurate allergy labels result in unnecessary avoidance of first-line beta-lactam antibiotics, increased use of broad-spectrum alternatives, and contribute to antimicrobial resistance and healthcare-associated infections. Data describing allergy-label documentation and de-labelling practices in the United Arab Emirates remain limited.

**Methods:**

This retrospective study examined temporal trends, documentation quality, and de-labelling patterns of penicillin and other beta-lactam allergy labels in EMRs from two tertiary care centres in Al Ain, United Arab Emirates, between 2015 and 2025. Extracted variables included age, sex, allergen class, adverse reaction type, reaction severity, duration of the documented allergy record, and de-labelling status.

**Results:**

A total of 35,134 penicillin allergy records and 6,317 other beta-lactam allergy records were identified. Adults and females accounted for most documented allergy labels. Documentation quality was suboptimal, with adverse reaction type and severity missing from the majority of records. De-labelling rates were higher for penicillin than for other beta-lactams but declined over time, decreasing from 16% in 2015 to approximately 10% in 2025. The annual proportion of documented penicillin allergy labels reached its lowest level in 2020 before increasing thereafter, whereas the proportion of other beta-lactam allergy labels remained relatively stable. De-labelling was more frequent among records documented for less than one year and among those classified as mild or involving cutaneous or gastrointestinal reactions, and less frequent among records documented for more than five years or classified as severe. Allergy-record duration reflected the time since first EMR documentation rather than the time since the original allergic reaction.

**Discussion:**

These findings demonstrate important gaps in allergy documentation and the persistence of incompletely characterized beta-lactam allergy labels within EMRs. Standardized documentation and structured, risk-stratified de-labelling strategies should be prioritized as part of antimicrobial stewardship programs to optimize beta-lactam utilization and help mitigate antimicrobial resistance.

## 1 Introduction

Penicillin allergy is the most documented drug allergy worldwide, with reported prevalence of 8%−15% in the general population and up to 15% among hospitalised patients (1–3). However, 90%−95% of individuals labelled as penicillin allergic are not truly allergic when evaluated using structured diagnostic protocols (4–6). Immediate IgE-mediated reactions represent the most clinically significant form of penicillin hypersensitivity because they may lead to rapid and potentially life-threatening manifestations such as anaphylaxis (7). Notably, IgE-mediated sensitivity declines over time, with approximately 80% of individuals losing reactivity after 10 years (8, 9). However, penicillin allergy labels frequently persist in electronic medical records, leading to unnecessary avoidance of first-line beta-lactam antibiotics and use of broader-spectrum alternatives. This practise is associated with increased risks of antimicrobial resistance, healthcare-associated infections, prolonged hospitalisation, and mortality, highlighting the need for systematic penicillin allergy assessment and de-labelling within antimicrobial stewardship programs (10–13).

Despite increasing recognition of penicillin allergy as a public health concern, important regional gaps remain in epidemiological data, diagnostic capacity, and de-labelling initiatives. Data on prevalence and management from the Middle East and Gulf Cooperation Council (GCC) countries remain limited compared with North America, Europe, and Australia (14, 15). Available studies highlight deficiencies in allergy documentation and evaluation. In Saudi Arabia, a national survey reported a self-reported prevalence of 9.5% (16). In Qatar, incomplete allergy documentation has been reported, with confirmatory testing recorded in only 0.21% of cases and some labelled patients subsequently tolerating penicillin (17–19). In Kuwait, a regional drug allergy registry reported confirmed drug allergy in 41.5% of evaluated patients, with beta-lactams accounting for 45.6% of confirmed reactions (20). Together, these findings indicate important gaps in the accuracy of allergy documentation and the availability of diagnostic and de-labelling services in Middle Eastern healthcare systems (21).

This setting offers a unique context to examine these issues due to its highly diverse population, with expatriates comprising over 80% of residents, and widespread adoption of electronic medical records (22, 23). However, no studies have systematically evaluated penicillin allergy documentation in this setting. International allergy societies recommend structured approaches to the evaluation and de-labelling of penicillin allergy (7, 24, 25). Evidence from systematic reviews also supports the safety of direct oral challenge in low-risk patients, with successful de-labelling in over 97% of cases and rare severe reactions (26–28). These findings support the feasibility of implementing structured penicillin allergy evaluation programs in the region.

However, studies that specifically assess penicillin and other beta-lactam allergy documentation in UAE electronic medical records are still limited. Therefore, this study aimed to examine the prevalence, trends, and quality of documentation of these allergy labels and to support future de-labelling plans in our healthcare setting.

## 2 Method

### 2.1 Study design

This retrospective study included all documented allergies recorded in the electronic medical record (EMR) allergy section through the pharmacy portal at two tertiary care centres in the United Arab Emirates. Both participating centres used the integrated Cerner Millennium electronic medical record system, which captures allergy documentation across clinical encounters. Drug allergy information was documented within the dedicated allergy module, which supports structured recording of the implicated drug or substance, reported reaction, allergy status, and additional clinical details (29). However, the completeness and quality of allergy documentation may depend on clinician documentation practises, including the use of structured fields and free-text documentation.

The study covered a 10-year period from January 1, 2015, to December 31, 2025, allowing assessment of long-term trends in annual allergy-label proportions and de-labelling practises, including potential impacts of the COVID-19 pandemic (2020–2021) on healthcare delivery and allergy documentation patterns. Ethical approval was granted by the Al Ain Region Human Research Ethics Committee (IRB reference: MF2058-2025-1160). The study involved retrospective analysis of de-identified records, and informed consent was waived by the ethics committee. This study was designed to evaluate penicillin allergy labelling as a modifiable target within antimicrobial stewardship programs.

### 2.2 Study population

The study included all patients registered in the hospital for any reason who had an encounter file opened and a documented penicillin or other β-lactam antibiotic allergy recorded in the allergy section of the electronic medical record at either participating institution. Eligible patients were identified through the Cerner EMR allergy module based on the presence of a documented penicillin or other β-lactam antibiotic allergy label during the study period. The allergy module captures both patient-reported and clinician-documented drug allergies, including the allergen name, reaction description, severity, date of documentation, and any subsequent updates or de-labelling actions. The EMR maintains a longitudinal allergy record across multiple patient encounters and care settings, allowing comprehensive assessment of allergy documentation over time. EMR-based allergy labels are widely used in antimicrobial stewardship research because they directly influence prescribing practises and clinical decision-support systems (1, 10).

### 2.3 Data variables

For each eligible patient, the following variables were extracted from from the Cerner Millennium EMR allergy module: a de-identified patient identifier, age, sex, allergen name and class (penicillin-class antibiotics or other β-lactams), date of allergy documentation, adverse reaction category, severity classification, duration of the allergy record, and de-labelling status and date (if applicable). Allergens were classified as penicillin-class antibiotics or other β-lactam antibiotics. Before statistical analysis, the extracted dataset was assessed for completeness, missing values, inconsistencies, and obvious data-entry errors. Records without a usable patient identifier or valid study year were excluded, although no such records were present in the final dataset. Age values recorded in days, weeks, or months were converted to years and checked against a valid range of 0–120 years. No statistical imputation was performed. Further details regarding data cleaning are provided in Supplementary Table S1. Allergy records and patient-level variables were coded using predefined categories. Age was classified as paediatric (<16 years) or adult (≥16 years), according to institutional definitions. Penicillin and other β-lactam labels were identified from the documented allergy type and substance. Cephalosporin generations was assigned using a drug-mapping table, with two mapping conflicts corrected before analysis. Documented reactions were grouped into the following standardised categories: (1) rash or other cutaneous reactions, (2) gastrointestinal symptoms, (3) respiratory reactions, (4) anaphylaxis, (5) severe cutaneous adverse reactions (SCAR), and (6) unknown or other reactions. These categories were based on reaction descriptions recorded in the EMR and were used for descriptive analysis only; they did not represent independent adjudication of the underlying allergic mechanism. Reaction severity was extracted directly from the EMR as documented and classified as mild, moderate, severe, or unknown, consistent with severity grading approaches used in allergy assessment literature (14, 30). Missing or blank reaction and severity fields were classified as unknown or undocumented because the assessment of documentation completeness was one of the study objectives. The duration of the documented allergy record was calculated from the date on which the allergy label was first recorded in the EMR to the date of data extraction and was categorised as <1 year, 1–5 years, >5 years, or unknown. This variable represented the duration of the documented allergy record rather than the time elapsed since the original allergic reaction because the date of the index reaction was not consistently available.

De-labelling was defined as a recorded allergy status indicating that the label had been removed. The method used for de-labelling, such as direct oral challenge, skin testing, or clinical history assessment, was not available in the dataset. For annual analyses, a patient-level variable was coded as present when at least one relevant record was documented during the corresponding calendar year. Each patient was counted once per calendar year within each relevant allergy class. The complete coding structure is presented in Supplementary Table S2. The allergy-record source variable was excluded from the analysis because of substantial missing data. No external validation against an independent data source was performed.

### 2.4 Statistical analysis

The main outcomes were the yearly proportion of documented penicillin and other beta-lactam allergy labels and the yearly de-labelling rates. Additional outcomes included the completeness of allergy documentation and the distributions of adverse reaction category, reaction severity, allergy-record duration, and de-labelling according to these characteristics.

Continuous variables were summarised using mean with standard deviation (SD) or median with interquartile range (IQR), as appropriate, and categorical variables were summarised as counts and percentages. Analyses were conducted at the patient-year level using unique medical record numbers to avoid double counting within a given calendar year. Analyses were conducted at the patient-year level using unique medical record numbers to prevent duplicate counting within the same calendar year. For each calendar year from 2015 through 2025, the denominator was the number of unique patients represented in the EMR allergy analytic extract. The numerator for each allergy class was the number of unique patients with at least one documented label in that class during the corresponding year. Repeated entries for the same patient within the same allergy class and calendar year were counted once. Patients with labels in more than one allergy class contributed to each relevant class-specific numerator. Annual de-labelling rates were calculated as the proportion of unique patients whose allergy label was removed among all patients with a documented allergy label in the same calendar year. De-labelling rates were calculated overall and separately for penicillin and other β-lactam allergies. Stratified analyses were performed to examine de-labelling rates according to allergy duration (<1 year, 1–5 years, >5 years), adverse reaction category (rash/cutaneous, gastrointestinal, respiratory, anaphylaxis, SCAR, unknown/other), and reaction severity (mild, moderate, severe, unknown). All analyses were performed using *R* (*R* Foundation for Statistical Computing, Vienna, Austria).

## 3 Results

### 3.1 Allergy profile characteristics

Over the 10-year study period (2015–2025), a total of 35,134 penicillin allergy records and 6,317 other beta-lactam allergy records were documented. Table 1 presents the demographic and clinical characteristics of these allergy records, stratified by time period (2015–2019 and 2020–2025) and allergy type (penicillin vs. other beta-lactams). The profile revealed a female predominance across both allergy types, with females comprising 65%−66% of penicillin allergy records. Adult patients represented the majority of cases, accounting for 76%−82% of all documented allergies, while paediatric patients comprised 18%−24% of records. A striking finding was the extremely high proportion of records with unknown or undocumented adverse reaction categories, affecting 74%−80% of all allergy labels. Similarly, severity information was missing in 87%−88% of cases, indicating substantial gaps in allergy documentation quality. Among documented adverse reaction categories, the most common categories included cutaneous and gastrointestinal symptoms. The duration of allergy records showed a notable temporal shift. The proportion of records with duration >5 years increased substantially from 18 to 19% in the 2015–2019 period to 41%−44% in the 2020–2025 period, reflecting both the accumulation of historical labels and the persistence of undocumented allergies in the healthcare system. The de-labelling rates were 23%−26% for penicillin allergies and 15%−17% for other beta-lactam allergies across the study period, indicating that approximately one-quarter of documented penicillin allergy labels were removed from the electronic medical record.

**Table 1:** Demographic and allergy profile characteristics of penicillin and other beta-lactam allergy records.

Characteristic	Category	Penicillin		Other beta-lactam antibiotics	
		2015–2019	2020–2025	2015–2019	2020–2025
Year of record *n* (%)		16,137 (4%)	18,997 (3%)	2,953 (0.7%)	3,364 (0.5%)
Age group^a^					
Adult	*n* (%)	13,228 (82.0)	15,436 (81.3)	2,332 (79.0)	2,573 (76.5)
	Mean age (SD), years	49.0 (20.4)	49.0 (19.2)	43.6 (21.8)	43.8 (20.3)
	Median age (IQR), years	47.0 (34.0–64.0)	48.0 (35.0–63.0)	41.0 (23.0–57.0)	42.0 (26.0–57.0)
Paediatric	*n* (%)	2,909 (18.0)	3,561 (18.7)	621 (21.0)	791 (23.5)
	Mean age (SD), years	11.2 (2.6)	9.6 (3.6)	11.3 (2.6)	9.9 (3.6)
	Median age (IQR), years	11.0 (9.0–13.0)	10.0 (7.0–13.0)	11.0 (9.0–14.0)	10.0 (7.0–13.0)
Gender, *n* (%)	Female	10,680 (66%)	12,263 (65%)	1,855 (63)	2,106 (63)
	Male	5,457 (34)	6,734(35)	1,098 (37)	1,258 (37)
Adverse reaction category, %	Cutaneous	20.53%	19.56%	16.80%	21.60%
	Respiratory	0.75%	0.84%	0.25%	0.40%
	Gastrointestinal	3.88%	3.75%	1.89%	2.25%
	Anaphylaxis	0.36%	0.40%	0.67%	0.86%
	SCAR^b^	0.13%	0.16%	0.12%	0.31%
	Other/unknown	74.34%	75.28%	80.26%	74.58%
Reaction severity,^c^ %	Mild	5%	5%	5%	6%
	Moderate	6%	6%	6%	5%
	Severe	1%	1%	2%	2%
	Unknown	88%	88%	87%	87%
Allergy duration,^d^ %	<1year	14%	12%	13%	12%
	1–5 years	34%	28%	33%	31%
	>5 years	19%	44%	18%	41%
	Unknown	33%	16%	35%	16%
Label removal,^e^ *n* (%)		4,123 (26)	4,289 (23)	491(17)	519 (15)

a Paediatric patients were younger than 16 years. Adults were aged 16 years or older based on prior local healthcare data systems. Age values recorded in months, weeks, or days were converted to years. Values are *n* (%), mean (SD), or median (IQR).

b Severe cutaneous adverse reaction.

c *Severe* reactions were identified based on entries documented in the allergy section of the Cerner electronic medical record system.

d Data are presented as *n* (%). Percentages may not total 100% because of rounding.

e Removed with no review/unknown.

### 3.2 Temporal trends in annual allergy-label proportions and de-labelling

**Figure 1:**
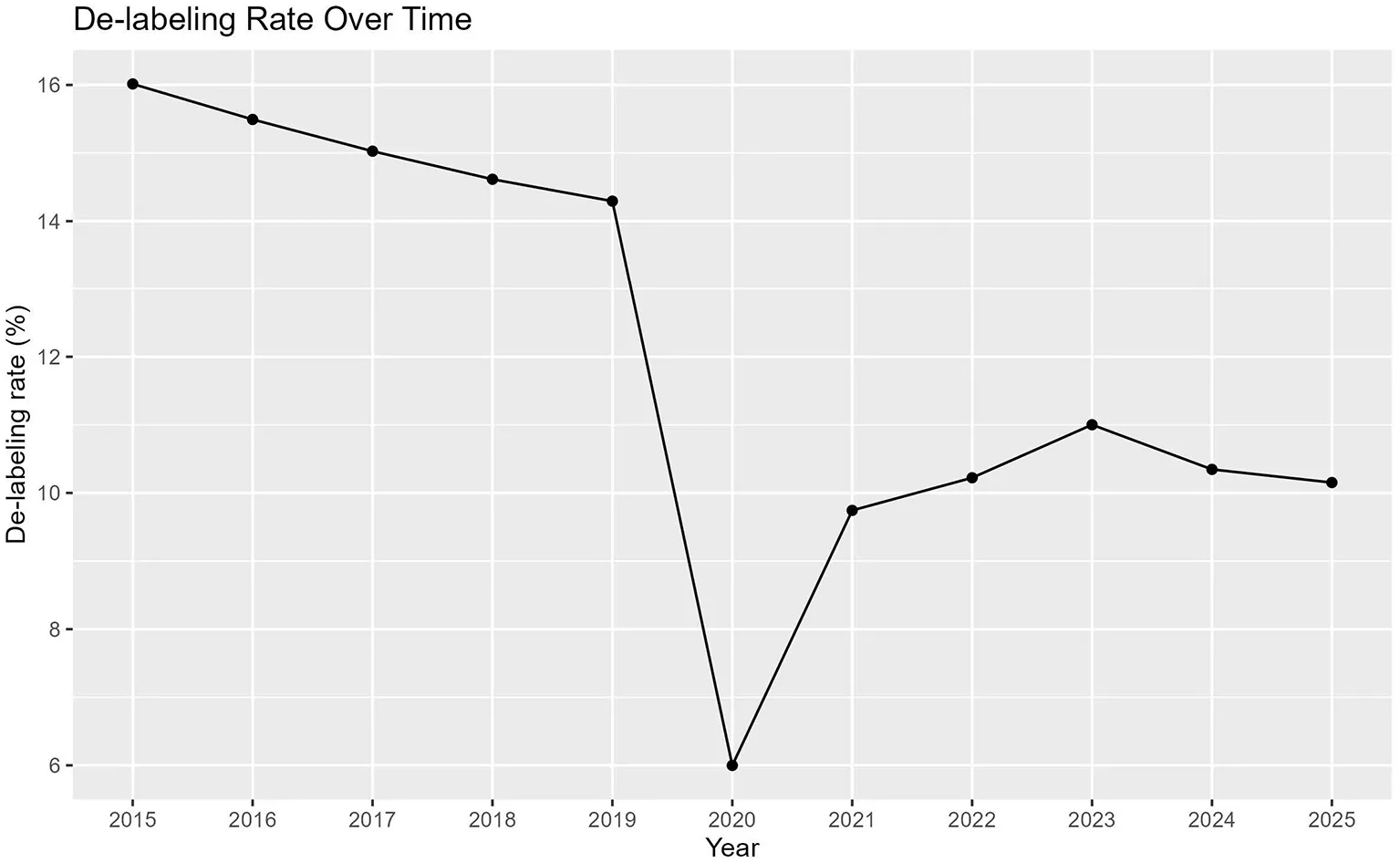
Penicillin allergy de-labelling trends (2015–2025). Annual proportion of documented penicillin allergy labels removed from the electronic medical record (EMR) (de-labelling defined as removal of the allergy entry from documentation) between 2015 and 2025 across the study institutions.

**Figure 2:**
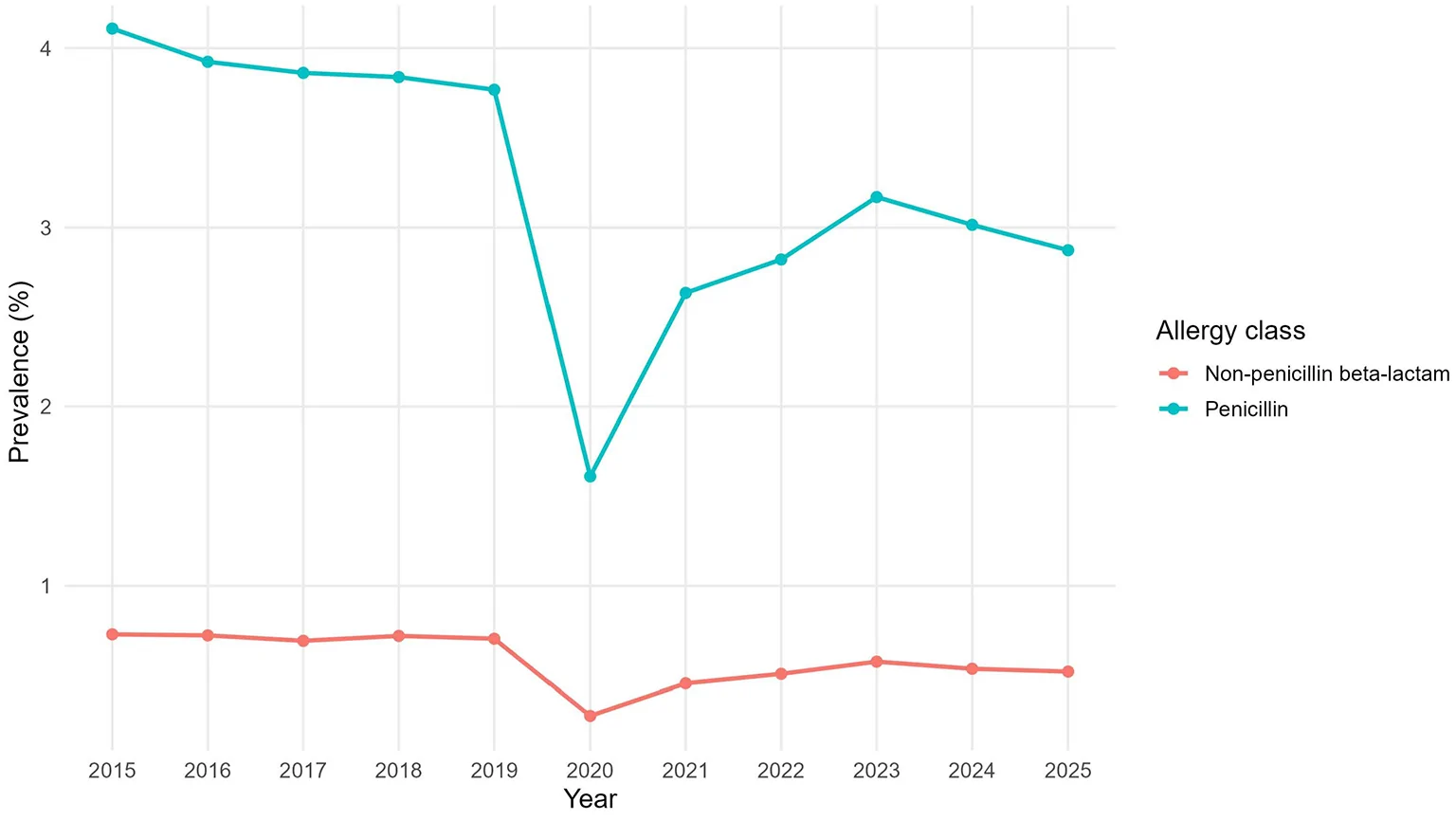
Annual trends in documented allergy-label proportions (2015–2025). Annual proportion of patients in the EMR allergy analytic extract with a documented penicillin or other beta-lactam allergy label. The denominator for each year was the number of unique medical record numbers in the annual analytic extract. The numerator was the number of unique patients with at least one label in the relevant allergy class during that year. Each patient was counted once per allergy class per year. Patients with both label types contributed to both series.

**Figure 3:**
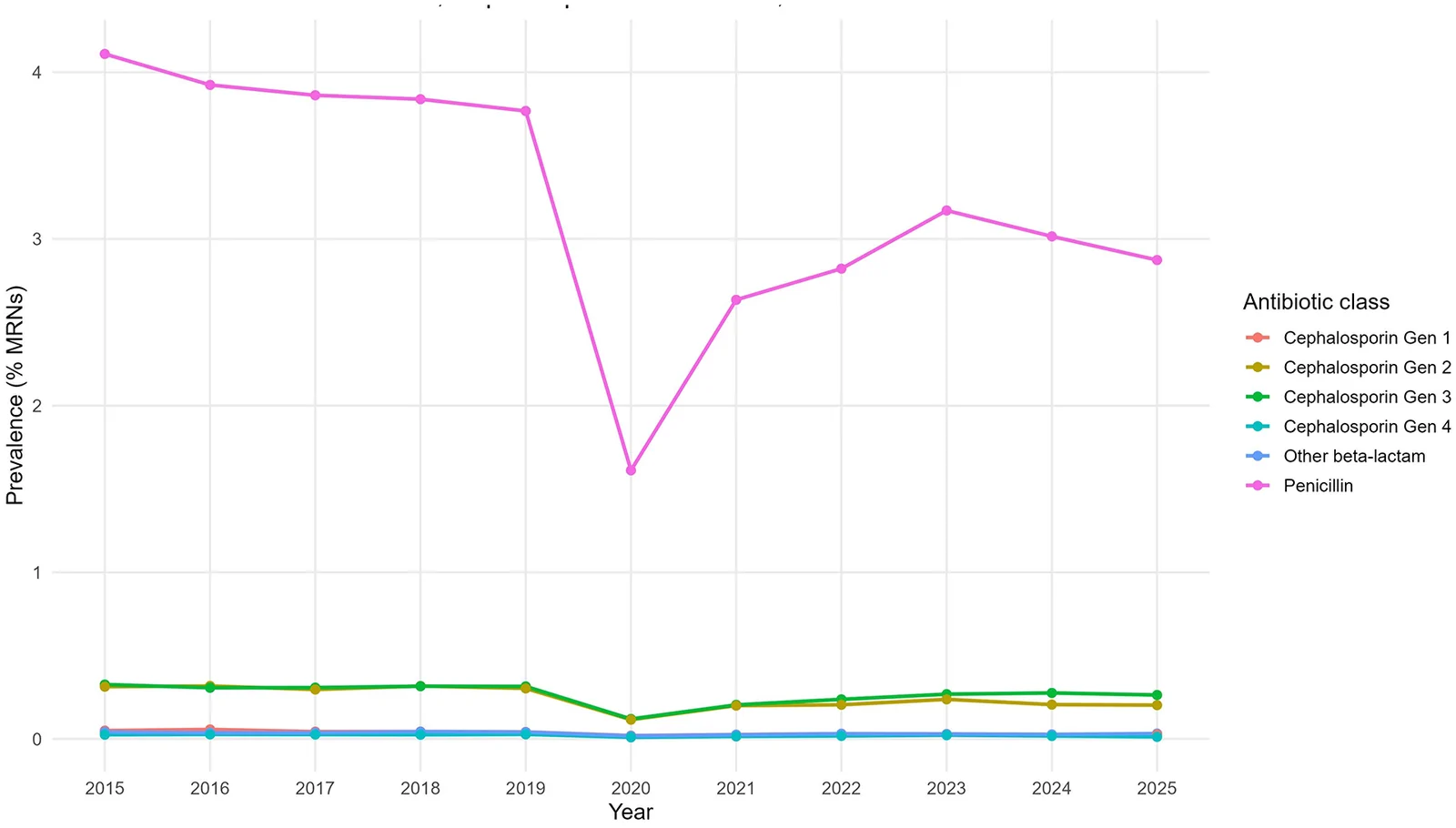
Annual trends in documented other β-lactam allergy-label proportions (2015–2025). Annual proportion of patients in the EMR allergy analytic extract with a documented penicillin, first- to fourth-generation cephalosporin, or other beta-lactam allergy label. No fifth-generation cephalosporin patient-year observations were identified. The denominator for each series was the number of unique medical record numbers in the annual analytic extract for that year. Each patient was counted once per antibiotic class per year and could contribute to more than one class.

Figure 1 illustrates the overall de-labelling rates from 2015 to 2025. De-labelling rates declined from 16% in 2015 to approximately 10% in 2025, with a notable drop observed in 2020. This decline may reflect changes in healthcare delivery patterns during the COVID-19 pandemic and subsequent shifts in allergy evaluation practises. Penicillin allergy annual proportions (Figure 2) demonstrated considerable variation over the study period, declining from 4.1% in 2015 to 1.6% in 2020 before increasing to 2.9% by 2025. These percentages represent annual proportions within the EMR allergy analytic extract and should be interpreted alongside the corresponding case counts and denominators presented in Table 2. In 2020, the number of unique patients with a penicillin allergy label increased from 3,119 to 3,416, while the annual denominator increased from 82,778 to 211,957. Therefore, the lower percentage in 2020 reflected the larger denominator rather than a reduction in the number of penicillin allergy cases. Other β-lactam allergy annual proportions (Figure 3) remained relatively stable at approximately 0.5%−0.7% throughout the study period. Annual case counts, denominators, and class-specific proportions are presented in Table 2 and Supplementary Table S3.

**Table 2:** Annual denominator and case count for documented penicillin and other beta-lactam allergy labels, 2015–2025.

Year	Annual denominator, *n*	Penicillin, *n* (%)	Other beta-lactam, *n* (%)	Both label types, *n*	Any beta-lactam, *n* (%)
2015	77,808	3,198 (4.11)	567 (0.73)	161	3,604 (4.63)
2016	83,991	3,296 (3.92)	607 (0.72)	169	3,734 (4.45)
2017	86,635	3,346 (3.86)	600 (0.69)	168	3,778 (4.36)
2018	82,791	3,178 (3.84)	596 (0.72)	157	3,617 (4.37)
2019	82,778	3,119 (3.77)	583 (0.70)	148	3,554 (4.29)
2020	211,957	3,416 (1.61)	583 (0.28)	142	3,857 (1.82)
2021	136,937	3,608 (2.63)	624 (0.46)	159	4,073 (2.97)
2022	125,735	3,548 (2.82)	639 (0.51)	166	4,021 (3.20)
2023	106,411	3,374 (3.17)	613 (0.58)	160	3,827 (3.60)
2024	99,953	3,014 (3.02)	536 (0.54)	142	3,408 (3.41)
2025	70,888	2,037 (2.87)	369 (0.52)	101	2,305 (3.25)

The annual denominator was the number of unique medical record numbers represented in the EMR allergy analytic extract. Repeated entries in the same class and calendar year were counted once. Patients with both label types contributed to both class-specific columns, so the penicillin and other beta-lactam columns should not be added.

### 3.3 De-labelling rates by duration of allergy record

**Figure 4:**
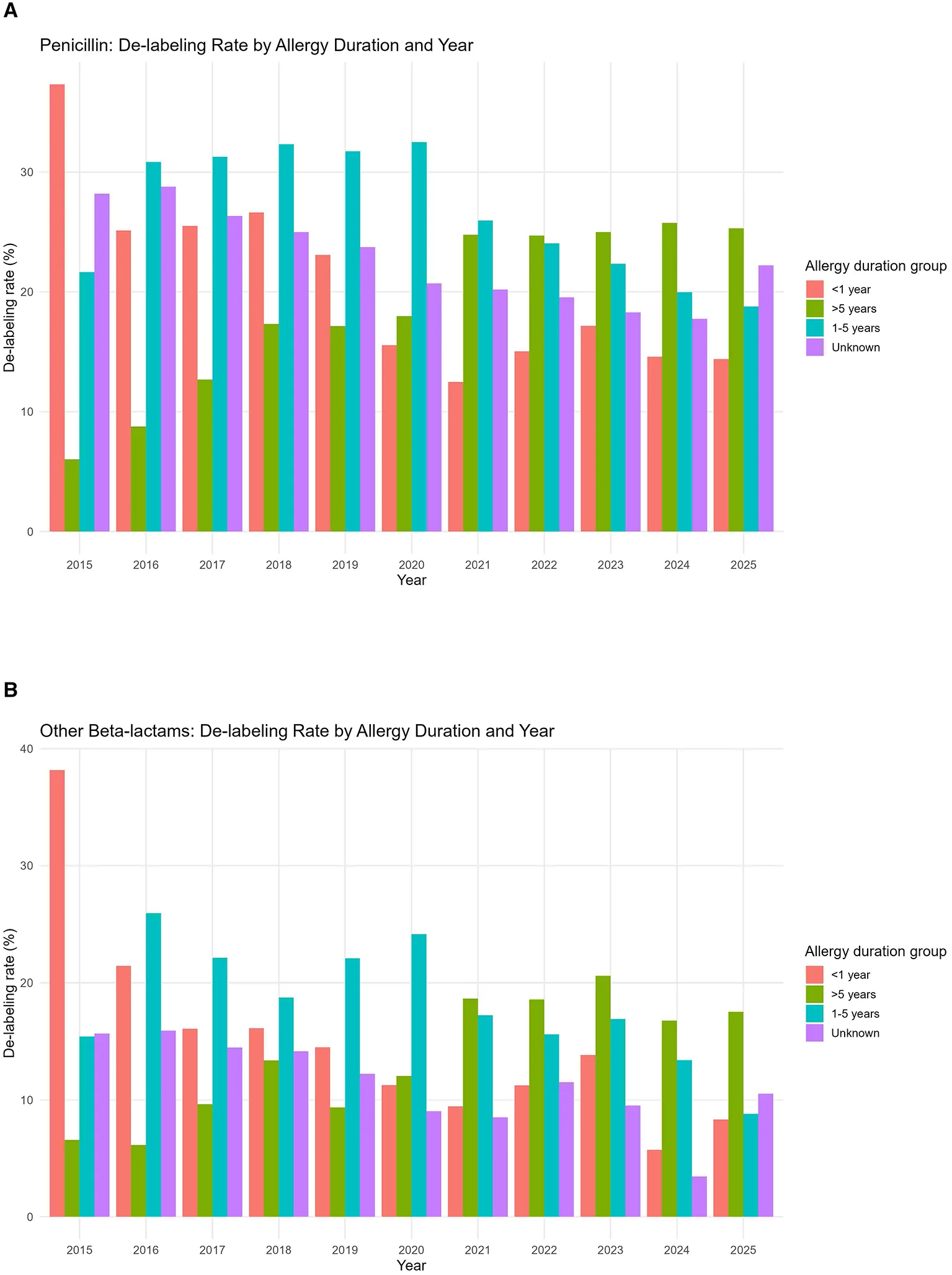
Beta-lactam allergy de-labelling rates by allergy duration (2015–2025). **(A)** Annual de-labelling rates for penicillin allergy labels stratified by reported allergy duration (<1 year, 1–5 years, >5 years, and unknown). **(B)** Annual de-labelling rates for other beta-lactam allergy labels stratified by reported allergy duration (<1 year, 1–5 years, >5 years, and unknown). De-labelling was defined as removal of the documented allergy label from the electronic medical record.

De-labelling rates varied according to allergy duration across the study period for both penicillin and other beta-lactam allergies (Figures 4A,B). For penicillin (Figure 4A), recently documented allergies (<1 year) generally demonstrated higher de-labelling rates, particularly in the earlier years of the study, although the 1–5-year duration group often showed comparable or higher rates in several years. In contrast, allergies of longer duration (>5 years) consistently exhibited lower de-labelling rates throughout the study period. Allergies of unknown duration followed patterns similar to long-standing labels.

A comparable trend was observed for other beta-lactam allergies (Figure 4B), with higher de-labelling rates seen among more recently documented allergies and lower rates among long-standing labels. Across both antibiotic groups, a progressive decline in de-labelling rates was observed over time across all duration categories, with the most marked reductions occurring in groups that initially demonstrated higher de-labelling rates. Overall, higher observed de-labelling rates were seen among more recently documented allergy labels, whereas lower rates were observed among long-standing labels, alongside a temporal decrease in de-labelling activity over the study period. These findings are descriptive and should not be interpreted as evidence that recently documented allergy labels are more appropriate for de-labelling.

### 3.4 De-labelling rates by adverse reaction category

**Figure 5:**
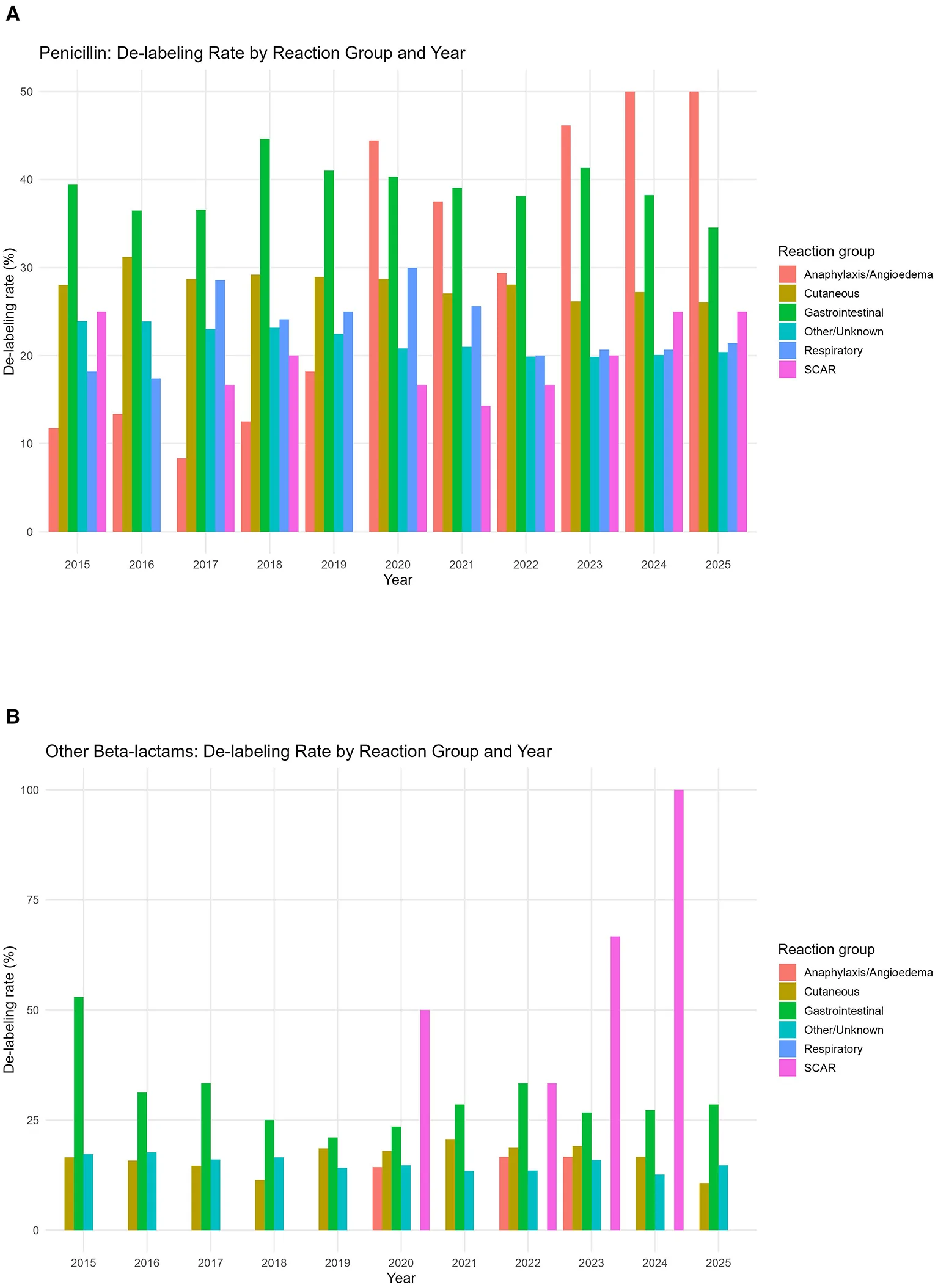
Beta-lactam allergy de-labelling rates by adverse reaction category (2015–2025). **(A)** Annual de-labelling rates for penicillin allergy labels stratified by documented adverse reaction category (anaphylaxis, cutaneous, gastrointestinal, respiratory, SCAR, and other/unknown). **(B)** Annual de-labelling rates for other beta-lactam allergy labels stratified by documented adverse reaction category (anaphylaxis, cutaneous, gastrointestinal, respiratory, SCAR, and other/unknown).

De-labelling rates varied by adverse reaction category for both penicillin and other beta-lactam allergies over the study period (Figures 5A,B). For penicillin (Figure 5A), cutaneous and gastrointestinal reactions showed consistently moderate to high de-labelling rates, while respiratory and other/unknown reactions were more variable. The proportion of allergy labels documented as anaphylaxis that were de-labelled increased over time, whereas SCAR remained lower overall. These findings are descriptive and should not be interpreted as supporting de-labelling of higher-risk allergy phenotypes. For other beta-lactams (Figure 5B), de-labelling rates were generally lower and more stable across most adverse reaction categories. Gastrointestinal and cutaneous reactions demonstrated modest but consistent rates, while SCAR showed marked variability with intermittent peaks. Although SCAR categories showed high rates in some years, the number of cases was very small (approximately 1–2 patients), and these estimates should be interpreted with caution and may reflect misclassification of reactions initially documented as severe. Anaphylaxis remained relatively low throughout. Overall, de-labelling rates differed by adverse reaction category, with more consistent patterns observed for non-severe reactions and greater variability among severe or less well-defined reactions.

### 3.5 De-labelling rates by severity

**Figure 6:**
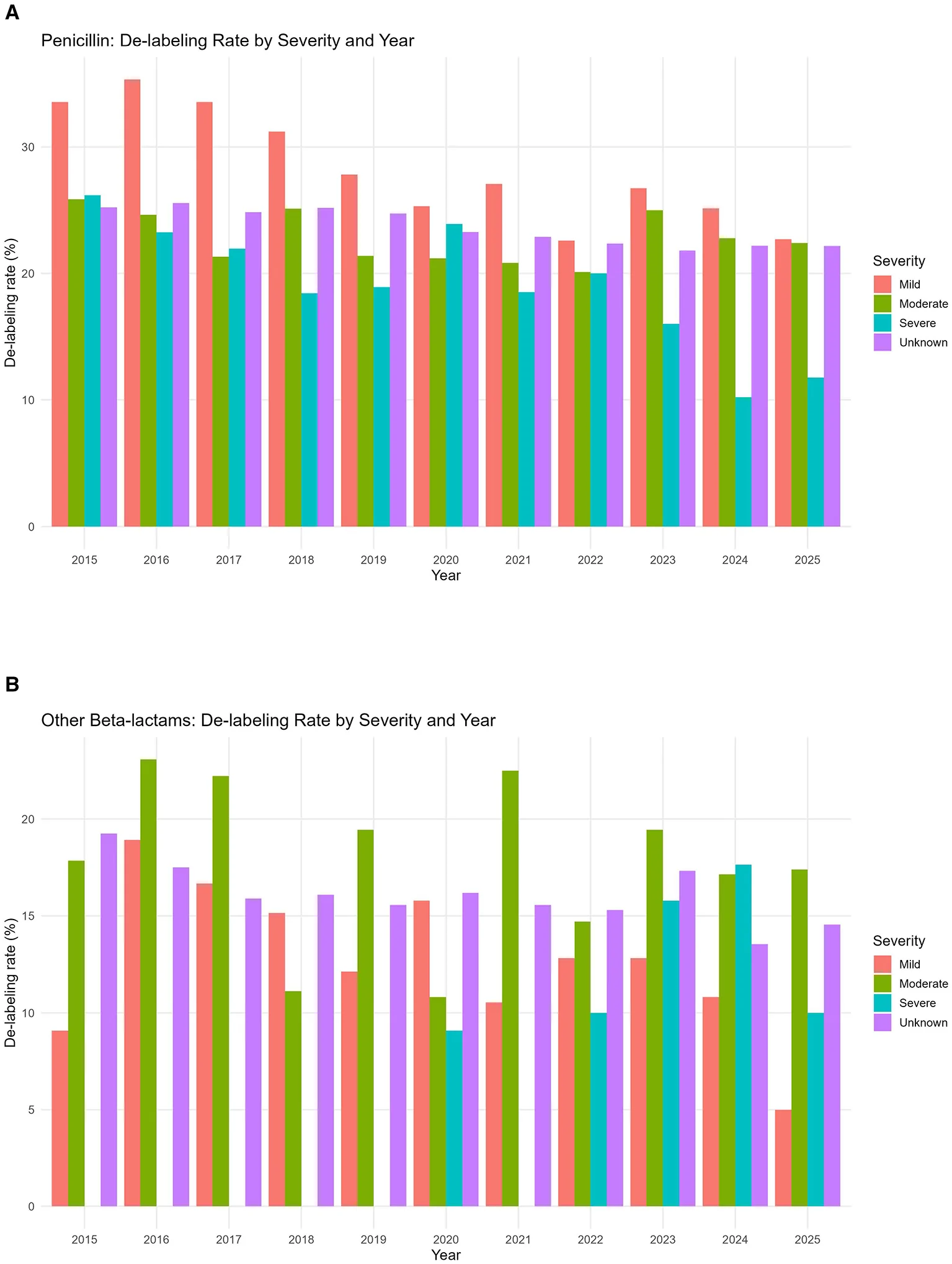
Beta-lactam allergy de-labelling rates by reaction severity (2015–2025). **(A)** Annual de-labelling rates for penicillin allergy labels stratified by documented reaction severity (mild, moderate, severe, and unknown). **(B)** Annual de-labelling rates for other beta-lactam allergy labels stratified by documented reaction severity (mild, moderate, severe, and unknown).

De-labelling rates differed according to reaction severity for both penicillin and other beta-lactam allergies over the study period (Figures 6A,B). For penicillin (Figure 6A), mild reactions consistently demonstrated the highest de-labelling rates, particularly in earlier years, with a gradual decline over time. Moderate reactions showed intermediate rates, while severe reactions were generally lower and more variable. Reactions of unknown severity followed patterns similar to moderate reactions. For other beta-lactams (Figure 6B), moderate reactions generally exhibited the highest observed de-labelling rates, while mild and unknown severity groups showed relatively stable but lower rates. Severe reactions consistently demonstrated the lowest de-labelling rates across most years. Across severity categories, higher observed de-labelling rates were seen in less severe reactions, whereas severe reactions consistently demonstrated lower rates across both antibiotic groups. These findings are descriptive and should not be interpreted as evidence that de-labelling decisions were based appropriately on documented reaction severity.

## 4 Discussion

This decade-long analysis provides important insights into penicillin and beta-lactam allergy documentation within the UAE. The observed annual proportions of documented penicillin allergy labels (1.6%−4.1%) were lower than the prevalence estimates of 8%−15% reported in North America and Europe and the global pooled prevalence estimate of 9.4% (14, 24, 31–33). However, these findings are not directly comparable because our annual proportions were calculated using patients represented in the EMR allergy analytic extract as the denominator rather than the entire hospital population.

Differences between our findings and international estimates may reflect variation in healthcare utilisation, antibiotic prescribing practises, antimicrobial stewardship infrastructure, patient reporting, allergy-history capture, and EMR documentation practises. Health literacy, access to allergy specialists, the availability of standardised guidance, and broader social determinants of health may also influence allergy recognition and documentation (34–41). However, because this study used routinely collected EMR data, the relative contribution of these factors could not be determined.

The higher proportion of documented allergy labels among women is consistent with previous literature describing sex differences in drug allergy reporting. These differences may reflect biological, pharmacological, and healthcare-related factors. However, because this study used routinely collected EMR data, the relative contribution of these factors to the observed sex difference could not be determined (42).

Despite the relatively low annual proportions of documented allergy labels, substantial deficiencies in documentation quality were identified. A large proportion of records lacked essential clinical details, with 74%−80% missing adverse reaction category and 87%−88% lacking severity information. These findings are consistent with international reports highlighting incomplete allergy documentation as a major barrier to appropriate antibiotic use (2, 14, 15, 43). In the absence of detailed reaction histories, clinicians often adopt a cautious approach and avoid beta-lactams, leading to increased use of broad-spectrum alternatives such as fluoroquinolones, carbapenems, and vancomycin. This practise has been associated with higher risks of antimicrobial resistance and healthcare-associated infections, including *Clostridioides difficile*, MRSA (methicillin-resistant *Staphylococcus aureus*), and vancomycin-resistant enterococci (2, 18–21, 24). The high proportion of missing severity data is particularly concerning, as severity assessment underpins contemporary risk-stratification strategies used in penicillin allergy de-labelling programs (25, 26).

De-labelling was documented in 23%−26% of penicillin allergy records. Although structured allergy assessment programs have reported that 90%−95% of evaluated patients can safely have an inaccurate penicillin allergy label removed (5, 44, 45), these figures are not directly comparable because our study measured routine EMR-documented de-labelling rather than the success of a formal testing program. The observed de-labelling rate may reflect incomplete documentation, limited access to specialist allergy services, and variation in local de-labelling practises.

The increasing proportion of long-standing allergy labels (>5 years), rising from approximately 18%−19% to 41%−44%, suggests progressive accumulation of historical and potentially inaccurate records. However, this pattern does not necessarily indicate an increase in longstanding true allergy because allergy-record duration was measured from the date of first EMR documentation rather than from the date of the original allergic reaction. The higher de-labelling rate among records documented for less than 1 year should therefore be interpreted cautiously. These records may include recently entered historical allergies, and the observed pattern may reflect documentation practises or non-standardised de-labelling processes rather than risk-based clinical decision-making. This finding differs from contemporary risk-stratification approaches, in which remote, benign allergy histories are generally considered more suitable for de-labelling than recent or severe reactions (25, 26).

From an antimicrobial stewardship perspective, the persistence of inaccurate penicillin allergy labels has important clinical and economic implications. Patients carrying such labels have been shown to experience longer hospital stays, higher readmission rates, increased treatment failures, and greater healthcare costs (46, 47). Avoidance of first-line beta-lactam therapy may also increase the risk of surgical site infections and other complications (48). Addressing inaccurate allergy labels therefore represents a key stewardship priority. Current de-labelling pathways typically involve a structured allergy history and risk stratification. Patients with low-risk histories may be eligible for direct oral challenge, whereas those with moderate- or high-risk histories generally require specialist assessment, which may include skin-prick testing, intradermal testing, oral challenge, or patch testing for selected delayed reactions (25, 26). Because the present study did not capture the methods used for de-labelling, the observed rates and patterns cannot be interpreted as evidence of safe, appropriate, or guideline-concordant practise. In particular, the higher de-labelling rates observed among records classified as anaphylaxis, moderate severity, or recently documented may reflect documentation practises or local workflows that could not be evaluated. Structured allergy evaluation and de-labelling initiatives can facilitate the use of optimal beta-lactam therapies, reduce reliance on broad-spectrum agents, and contribute to efforts to limit antimicrobial resistance (49–51). Antimicrobial stewardship teams, including clinical pharmacists, can facilitate implementation of these risk-stratified de-labelling pathways by identifying suitable patients, coordinating specialist referral when indicated, and promoting appropriate beta-lactam use while maintaining patient safety (49–52). These findings highlight penicillin allergy labelling as a critical and addressable target for antimicrobial stewardship programs, particularly in settings where structured de-labelling pathways are not yet widely implemented.

### 4.1 Limitations

Several limitations should be considered when interpreting these findings. First, this analysis relied on electronic health record data, which may be incomplete or inconsistently recorded and likely contributed to the high proportion of missing information on adverse reaction category and severity. The EMR allergy module included both patient-reported and clinician-documented allergies, which may differ in diagnostic accuracy. Because information on the source of the allergy record was frequently missing, we were unable to assess whether the findings differed according to the source of documentation. Furthermore, the duration of the documented allergy label was based on the time the allergy label was recorded in the electronic medical record and did not necessarily reflect the time elapsed since the original allergic reaction, as the date of the index reaction was not consistently documented. In addition, the increase in allergy labels with a documented duration of >5 years may partly reflect accumulation of previously recorded allergy labels over time rather than a true temporal change in allergy epidemiology. Second, the dataset did not capture details on the specific methods used for allergy de-labelling (e.g., direct oral challenge, skin testing, or clinical history-based assessment), limiting evaluation of the appropriateness of de-labelling practises. Consequently, the appropriateness and safety of the observed de-labelling practises could not be evaluated. Third, other β-lactam antibiotics were analysed as a single group in accordance with the predefined study classification. Consequently, separate patterns of allergy documentation and de-labelling among cephalosporins and carbapenems were not assessed, although such analyses may provide additional insights relevant to antimicrobial stewardship. Fourth, changes observed in 2020 may have coincided with disruptions in healthcare utilisation and documentation during the COVID-19 pandemic (41). However, because no formal interrupted time-series or comparative analysis was performed, these changes cannot be attributed specifically to the pandemic. Fifth, downstream clinical outcomes, including antibiotic prescribing patterns, infection-related outcomes, and adverse drug reactions following de-labelling, were not assessed. Finally, this study was conducted in two tertiary hospitals in the Emirate of Abu Dhabi. Although healthcare organisations across the UAE may use different underlying electronic medical record platforms and local documentation workflows (53, 54), differences in EMR functionality and the tertiary-care setting of the participating centres should be considered when interpreting the generalisability of these findings to non-tertiary hospitals, primary care settings, and other healthcare facilities across the UAE.

### 4.2 Future directions

Future research should focus on improving the quality of allergy documentation and expanding safe, scalable penicillin allergy de-labelling strategies within the UAE healthcare system. Prospective studies evaluating risk-stratified approaches, including validated tools such as PEN-FAST, may help determine whether low-risk patients can be safely assessed outside specialist settings (25, 26, 55). Implementation of structured electronic health record documentation and integration of pharmacist-led antimicrobial stewardship programs may further support systematic de-labelling initiatives (32). In addition, studies assessing the clinical and economic impact of penicillin allergy labels, as well as barriers to implementation across healthcare settings, are needed to inform sustainable strategies. Building on these findings, we are currently conducting a prospective study to evaluate a risk-stratified penicillin allergy de-labelling pathway, with the aim of assessing its feasibility, safety, and clinical impact.

## 5 Conclusion

The annual proportions of documented penicillin allergy labels in the UAE allergy analytic extract were lower than prevalence estimates reported in Western populations; however, these measures are not directly comparable because of differences in study denominators. Documentation quality was suboptimal, with most records lacking key information on adverse reaction category and severity. De-labelling occurred in a minority of cases and declined over time, while long-standing labels (>5 years) accumulated within the electronic medical record. These findings highlight significant gaps in allergy documentation and suggest that persistent penicillin allergy labels may represent an under-recognised barrier to optimal antibiotic use. Implementation of standardised documentation practises and structured, risk-stratified de-labelling strategies should be prioritised as core components of antimicrobial stewardship programs to improve antibiotic selection and mitigate antimicrobial resistance.

## Data Availability

Derived data are available from the corresponding author upon reasonable request. Requests to access these datasets should be directed to FA, famarzooqi@uaeu.ac.ae.
