# Supplementary material for: Penicillin allergy documentation in the UAE: 10-year trends and stewardship implications
Source: Front Med (Lausanne). 2026 Aug 6;13:1891395. doi: 10.3389/fmed.2026.1891395 (PMC13490189; doi:10.3389/fmed.2026.1891395)
Supplement: Supplementary file 1 [file Table_1.pdf]

Supplementary Table S1. Data management and missing-data summary

Item	Result	How it was handled
Source rows loaded	7,211,118	Rows read before patient identifier and year checks.
Beta-lactam source rows	376,658	Rows identified as penicillin or another beta-lactam before patient-year deduplication.
Missing or blank patient identifier	0	Records without a usable identifier would be excluded. None were found.
Invalid or out-of-range study year	0	Records outside 2015-2025 or without a usable year would be excluded. None were found.
Age missing or invalid	0 (0.00%)	Ages in months, weeks, or days were converted to years. Values <0 or >120 years were treated as missing. No imputation was used.
Reaction description missing or blank	267,890 (71.12%)	Excluded from analysis.
Severity missing or blank	333,869 (88.64%)	Excluded from analysis.
Allergy duration missing or blank	98,347 (26.11%)	Excluded from analysis.
Statistical imputation	None	Analyses used available valid values.

Missing-data percentages in this table use the 376,658 beta-lactam source rows as the denominator. The annual prevalence and age analyses used patient-year observations.

Supplementary Table S2. Coding structure for allergy records and patient-level variables

Analysis variable	Source field	Coding used in the analysis
Patient identifier	MRN or equivalent patient identifier	Converted to character. Missing or blank identifiers were excluded.
Calendar year	Year field or worksheet name	Converted to integer and restricted to 2015-2025.
Age	Age field	Converted to years. Months were divided by 12; weeks were multiplied by 7/365.25; days were divided by 365.25.
Age group	Derived from age in years	Paediatric <16 years; adult >=16 years; missing or invalid age Unknown.
Sex	Gender or sex field	Standardised as Male, Female, or Unknown.
Penicillin label	Allergy type and substance	Dictionary-based text matching for penicillin and penicillin-class names.
Other beta-lactam label	Allergy type and substance	Dictionary-based matching for cephalosporins, carbapenems, monobactams, and general beta-lactam terms; penicillin rows were excluded from this row-level class.
Figure 3 antibiotic class	Substance mapping workbook and broad allergy-class flags	The penicillin group used the same penicillin flag as Figure 2. Cephalosporin generation was taken from the mapping field or class tag. Cefdinir/Omnicef was assigned to generation 3 and cefprozil/Cefzil to

		generation 2. Remaining non-penicillin beta-lactams were grouped as Other beta-lactam.
Reaction group	Free-text reaction description	First matching group in this order: SCAR; anaphylaxis/angioedema; respiratory; gastrointestinal; cutaneous; other/unknown.
Severity	Severity field	Retained as documented.
Allergy duration	Extracted allergy-duration field	Values with stated units were converted to years and grouped as <1 year, 1-5 years, >5 years, or Unknown.
De-labelling status	Allergy status field	Text indicating removed, deleted, resolved, cleared, cancelled/canceled, or de-labelled was coded as removed.
Annual denominator	Derived patient-year table	Unique MRNs represented in the annual EMR allergy analytic extract
Annual numerator	Derived patient-year class flags	Unique MRNs with at least one label in the relevant class during that calendar year.

Supplementary Table S3. Annual case counts and proportions by beta-lactam class, 2015-2025

**Year**	**Denominator**	**Penicillin**	**Ceph G1**	**Ceph G2**	**Ceph G3**	**Ceph G4**	**Ceph G5**	**Other beta-lactam**
2015	77,808	3,198 (4.11)	39 (0.05)	244 (0.31)	254 (0.33)	20 (0.03)	0 (0.00)	32 (0.04)
2016	83,991	3,296 (3.92)	48 (0.06)	267 (0.32)	258 (0.31)	23 (0.03)	0 (0.00)	31 (0.04)
2017	86,635	3,346 (3.86)	38 (0.04)	257 (0.30)	267 (0.31)	23 (0.03)	0 (0.00)	32 (0.04)
2018	82,791	3,178 (3.84)	37 (0.04)	263 (0.32)	262 (0.32)	21 (0.03)	0 (0.00)	36 (0.04)
2019	82,778	3,119 (3.77)	35 (0.04)	251 (0.30)	261 (0.32)	23 (0.03)	0 (0.00)	34 (0.04)
2020	211,957	3,416 (1.61)	43 (0.02)	245 (0.12)	254 (0.12)	21 (0.01)	0 (0.00)	38 (0.02)
2021	136,937	3,608 (2.63)	36 (0.03)	274 (0.20)	280 (0.20)	21 (0.02)	0 (0.00)	34 (0.02)
2022	125,735	3,548 (2.82)	40 (0.03)	258 (0.21)	299 (0.24)	23 (0.02)	0 (0.00)	39 (0.03)
2023	106,411	3,374 (3.17)	32 (0.03)	253 (0.24)	286 (0.27)	24 (0.02)	0 (0.00)	31 (0.03)
2024	99,953	3,014 (3.02)	27 (0.03)	206 (0.21)	276 (0.28)	18 (0.02)	0 (0.00)	27 (0.03)
2025	70,888	2,037 (2.87)	19 (0.03)	144 (0.20)	187 (0.26)	9 (0.01)	0 (0.00)	23 (0.03)

Values are unique patients, n (%). The denominator was the number of unique medical record numbers in the annual EMR allergy analytic extract. A patient could contribute to more than one antibiotic class in the same year. No fifth-generation cephalosporin patient-year observations were identified.
